# A Conserved Transcriptional Signature of Delayed Aging and Reduced Disease Vulnerability Is Partially Mediated by SIRT3

**DOI:** 10.1371/journal.pone.0120738

**Published:** 2015-04-01

**Authors:** Jamie L. Barger, Rozalyn M. Anderson, Michael A. Newton, Cristina da Silva, James A. Vann, Thomas D. Pugh, Shinichi Someya, Tomas A. Prolla, Richard Weindruch

**Affiliations:** 1 LifeGen Technologies LLC, Madison, Wisconsin, United States of America; 2 Department of Medicine, SMPH, University of Wisconsin, Madison, Wisconsin, United States of America; 3 Geriatric Research, Education and Clinical Center, William S. Middleton Memorial Veterans Hospital, Madison, Wisconsin, United States of America; 4 Departments of Statistics and of Biostatistics and Medical Informatics, University of Wisconsin, Madison, Wisconsin, United States of America; 5 Departments of Genetics and Medical Genetics, University of Wisconsin, Madison, Wisconsin, United States of America; University of Valencia, SPAIN

## Abstract

Aging is the most significant risk factor for a range of diseases, including many cancers, neurodegeneration, cardiovascular disease, and diabetes. Caloric restriction (CR) without malnutrition delays aging in diverse species, and therefore offers unique insights into age-related disease vulnerability. Previous studies suggest that there are shared mechanisms of disease resistance associated with delayed aging, however quantitative support is lacking. We therefore sought to identify a common response to CR in diverse tissues and species and determine whether this signature would reflect health status independent of aging. We analyzed gene expression datasets from eight tissues of mice subjected to CR and identified a common transcriptional signature that includes functional categories of mitochondrial energy metabolism, inflammation and ribosomal structure. This signature is detected in flies, rats, and rhesus monkeys on CR, indicating aspects of CR that are evolutionarily conserved. Detection of the signature in mouse genetic models of slowed aging indicates that it is not unique to CR but rather a common aspect of extended longevity. Mice lacking the NAD-dependent deacetylase SIRT3 fail to induce mitochondrial and anti-inflammatory elements of the signature in response to CR, suggesting a potential mechanism involving SIRT3. The inverse of this transcriptional signature is detected with consumption of a high fat diet, obesity and metabolic disease, and is reversed in response to interventions that decrease disease risk. We propose that this evolutionarily conserved, tissue-independent, transcriptional signature of delayed aging and reduced disease vulnerability is a promising target for developing therapies for age-related diseases.

## Introduction

Aging is the most significant risk factor for a range of diseases, including many cancers, neurodegeneration, cardiovascular disease, and diabetes. It is common to all animals [1], however the factors underlying age-related disease vulnerability are not known [2]. Caloric restriction (CR) without malnutrition delays aging in diverse species [3], including non-human primates [4], and therefore offers a unique perspective on identifying fundamental mechanisms of disease vulnerability. Previous studies indicate that CR acts in parallel across tissues: it prevents or attenuates the majority of age-associated changes in gene expression [5–8] and it delays the onset of multiple age-associated diseases and disorders that are of distinct tissue origin [9]. Together, existing data suggest that delayed aging via CR is a tissue-coordinated response with an evolutionarily conserved mechanism.

Additional insight into mechanisms of delayed aging and decreased risk of disease may be gleaned from studies of long-lived mice [10] and from pharmaceutical and lifestyle interventions. Ames and Snell dwarf mice have genetic mutations in genes that attenuate endocrine signaling from the pituitary gland and lifespan extension of ~50% is observed for each of these mouse strains [11, 12]. The “little” mouse has a mutation in the growth hormone releasing hormone receptor resulting in low levels of circulating growth hormone and lifespan extension of ~25% [12]. GHRKO mice, also known as Laron mice, have a disruption in the *Ghr* gene that encodes the growth hormone receptor/binding protein, and exhibit lifespan extension of ~20 or 40% for females and males, respectively [13]. Weight loss and treatment with thiazolidinediones induce multiple hallmarks of CR including increased insulin sensitivity, activation of mitochondrial metabolism and reduced inflammation [14, 15]. Consumption of the polyphenol resveratrol mimics the metabolic and anti-inflammatory action of CR in metabolically compromised subjects [16, 17].

We wished to examine if there are quantitative similarities in the mechanisms of delayed aging by CR, and if such patterns are also observed in other studies of delayed aging and decreased risk of disease. Previous analyses have identified individual genes that are regulated across tissues by CR in mice [18–20], however a gene-level approach may fail to detect common mechanisms of delayed aging due to tissue specificity in transcription (different genes may regulate the same pathway in different tissues [21]. Additional limitations of gene-level approaches include discrepancies in transcript representation across technical platforms and uncertainty in gene homology/orthology between species. Here we report the results of an analysis designed to test if delayed aging is mediated by a set of shared gene functional classes. We first identified a response to CR that is common across eight mouse tissues and found that this pattern is quantitatively recapitulated in flies, rats and primates subjected to CR, as well as long-lived mouse genetic models. Mice lacking SIRT3 fail to induce aspects of this response when subjected to CR. Finally, the inverse of the delayed aging signature is observed in conditions that increase risk of disease, whereas treatments for metabolic disease induce the delayed aging signature.

## Materials and Methods

### Data selection

We define CR as a regimen of reduced calorie intake in the absence of malnutrition with demonstrated ability to delay aging and the onset of age associated disease. In order to maintain consistency in transcript identification across studies, we only used microarray datasets that were generated using Affymetrix platforms. Because dietary regimens such as every other day feeding may not extend lifespan [22], we excluded studies where actual calorie intake was not documented (e.g., group-housed CR animals, ad lib vs. CR, or every other day ad lib feeding). Similarly, we also excluded those studies of restricted food intake where the nutritional regimen or duration of CR had not been previously demonstrated to delay aging (e.g., one week of a calorie restricted diet). If there were multiple datasets for the effect of CR in a tissue, we selected the study that used the most recent microarray platform or where the raw, unprocessed gene expression data (CEL files) were available. One exception was in mouse heart where a newer dataset was available, however the same individual mice in the newer study were also studied for gene expression in cerebral cortex. Thus, an alternate dataset for gene expression in mouse heart was used for purposes of statistical independence. A listing of the datasets analyzed in our study can be found in S1 Table.

### Animal care

Experimental details for publicly-available datasets are described in their original publications (see S1 Table) and a thorough description regarding the housing and feeding regimen for control and CR animals is described in detail elsewhere [23]. Five previously unpublished datasets were analyzed in the current study.

Mouse studies were performed at the William S. Middleton Memorial Veteran’s Hospital (AAALAC registration number VA-044) and all studies were approved by the IACUC at the William S. Middleton Memorial Veteran’s Hospital. Mice were euthanized by cervical dislocation and no tissues were collected from mice prior to euthanization. For the previously unpublished gene expression datasets, sample sizes and duration for which mice were subjected to a calorie restricted (CR) diet are as follows (animals were randomly assigned to either control or CR groups and microarray experiments were performed with equal numbers of Control and CR animals on each day):

1. Mouse hippocampus: male C57BL/6Hsd mice were subjected to CR from 12 to 25 months of age (*n* = 5 control and *n* = 5 CR mice per group);
2. Mouse kidney: male C57BL/6Hsd mice were subjected to CR from 12 to 25 months of age (*n* = 5 control and *n* = 5 CR mice per group);
3. Mouse gastrocnemius muscle in wild type and Sirt3^-/-^ mice: male mice were subjected to CR from 2 to 12 months of age (*n* = 4 control and *n* = 4 CR mice per group).

Studies of rhesus monkeys were performed at the Wisconsin National Primate Research Center (WNPRC) at the University of Wisconsin Madison (Laboratory Animal Welfare Public Health Service Assurance Number: A3368–01) and were approved by the University of Wisconsin Graduate School IACUC. No animals were euthanized in order to collect samples used in these studies.

Rhesus monkeys were maintained in a specific pathogen free facility and were housed individually to regulate food intake. Animals were fed a semi-purified, nutritionally fortified, low fat diet containing 15% protein and 10% fat. The CR regimen was implemented as follows: after pre-assignment clinical testing, all animals underwent a three-month lead-in period of transition to the study semi-purified diet. Once acclimated, a three-month baseline period of food intake for each individual was measured. Following the baseline period, animals were randomized based on age, body weight, and food intake to either CR or Control group and food allotments for the CR animals were restricted by 10% per month for three months to reach the desired level of 30% restriction. Food intake was measured daily for all animals on this project. Each animal was given a pre-measured, individualized, allotment of food in the morning (approximately 7–9am). In the afternoon (approximately 3–5pm), any food remaining in the feeder bin was removed and a healthy snack was provided in the form of a piece of fruit. Weight of uneaten food was subtracted from original food allotment to determine daily food intake. The WNPRC ensures the enrichment of the monkey’s environment by providing materials that increase activity and provide an outlet for the animals’ behavior instincts.

As part of the study design, animals were treated for presenting conditions. For example, diabetic monkeys received insulin sensitizers or insulin and animals with diverticulosis receive fiber supplements. For biopsy collection (in this study, vastus lateralis muscle and peripheral blood mononuclear cells—PBMCs), the animals were given buprenorphine (0.01–0.03 mg/kg, IM) in the morning immediately preceding all biopsies and again that afternoon. Further post-operative analgesia (acetaminophen up to 6 mg/kg, PO, ketoprofen up to 6 mg/kg, IM, or buprenorphine 0.01–0.03 mg/kg, IM) were administered as needed, per a veterinarian’s recommendation, to alleviate discomfort. Signs of discomfort include, but are not limited to lack of appetite and limited mobility. At least twice daily, animal care staff observed all animals for signs of pain, illness, and stress by noting appetite, stool, typical behavior, physical condition, etc. If any of the above parameters were found to be unacceptable, a member of the WNPRC veterinary staff was notified and their recommendations followed. When clinical signs were present, diagnostics were undertaken in an attempt to reach a definitive diagnosis. For animals diagnosed with a treatable disease, it was determined whether or not administration of treatment would maintain the animal’s quality of life to an acceptable degree. Quality of life is determined by evaluation of a) food intake, b) ability to maintain body weight & condition, c) locomotive ability and the ability to manipulate objects/food normally, and d) quality of daily interactions with personnel. Each animal has his or her own “normal” based on that specific animal’s personality and this individualized “normal” is used as the basis of comparison. No rhesus monkeys were euthanized for the current studies. For the previously unpublished gene expression datasets, sample sizes and duration for which animals were subjected to a calorie restricted (CR) diet are as follows:

1. Rhesus monkey vastus lateralis muscle: at the time of tissue collections, control animals (27–30 years old) and CR animals (27–32 years old) had been subjected to CR for 18.5 years (n = 5 control and n = 8 CR monkeys per group);
2. Rhesus monkey PBMCs: at the time of blood collection, control animals (24–27 years old) and CR animals (24–30 years old) were subjected to CR for 16 years (n = 7 control and n = 7 CR monkeys per group).

### Microarray data processing

Because two studies were only available in MAS5 format, we applied the MAS5 algorithm to generate signal intensity data from CEL files for all studies. Whereas different algorithms may reveal subtle differences in the fold change in expression of individual genes, a preliminary analysis indicated that both MAS5 and RMA algorithms generated identical results at the level of gene functional classes. For each study, microarray signal intensity data were first filtered to a set of unique transcripts with known Entrez Gene IDs. Briefly, we removed probe sets that were either not annotated with a known Entrez Gene ID or where a probe set was annotated with multiple transcripts; for the latter, the probeset having the largest average signal intensity (across all arrays within a study) was selected for gene set analysis. All gene expression datasets will be uploaded to the NCBI Gene Expression Omnibus and accession numbers will be provided after peer review.

### Gene set analysis

We used Parametric Analysis of Gene set Enrichment (PAGE, ref. [24]) to derive a *Z*-score for each GO term that reflects the treatment-induced modulation of that pathway. PAGE utilizes predefined functional classes as defined by the Gene Ontology (GO) consortium (http://geneontology.org) and tests for a treatment effect across the category as a whole without the requirement for significant changes in individual genes. We considered GO terms that were annotated with at least 10 but not more than 1000 genes at the time of the analysis. To construct a multi-term signature of CR in mouse tissue, we applied term-specific one-sample *t*-test and used two filters to identify the final list of terms modulated by CR: first, the Benjamini-Hochberg corrected *t*-test *p*-value had to be smaller than 0.00285; second, the median *Z*-score (over tissues) ≥ 2.58 or ≤ -2.58 (corresponding to a nominal set *p*-value of 0.001). For this calculation we started with *n* = 4,949 GO terms having *Z*-scores on at least two tissues. To assess the statistical significance of the observed (Spearman) correlation between median *Z*-scores computed separately within the mouse CR tissues and the non-mouse CR tissues (as well as between mouse CR and long-lived mouse models), we considered both permutation sampling and bootstrap sampling schemes. Permutation schemes aim to assess the null hypothesis that mouse CR and non-mouse CR (or mouse CR and mouse genetic model) tissues yield independent functional profiles; they reconstruct pseudo-data under that hypothesis and report the expected null variation in the correlation statistic. On the other hand, bootstrap schemes aim to assess the sampling distribution of the measured correlation, and thus provide confidence intervals for the underlying correlation. For both schemes we were concerned that sampling distributions could be affected by the known overlap of functional categories represented in the GO hierarchy. To address this, we invoked both the standard permutation/bootstrap sampling, which ignores category overlap, and custom-built samplers that accommodate the overlap among genes within GO terms via Gaussian models and a formula for the overlap-induced correlation of functional-category *Z*-scores [25]. The four schemes were:

1. **permutation**: shuffle the association between components of vectors mouse CR Z-score and non-mouse CR Z-score; re-compute the correlation on each permuted set;
2. **pair bootstrap**: randomly sample GO terms with replacement from the full collection; each term gives a pair of Z scores (one from mouse CR, one from non-mouse CR); re-compute the correlation on each bootstrap sample;
3. **model-based permutation**: using the predicted null correlation: set overlap / (Set size1 × Set size2)^0.5^, simulating mean of zero but with correlated Gaussian vectors, one for mouse CR and one for non-mouse CR, using the Cholesky decomposition to induce term-by-term correlation; re-compute the mouse/non-mouse correlation on each simulated data set;
4. **model-based bootstrap**; like 3, but use a non-zero mean vector computed by component-wise averaging of mouse CR Z-score and non-mouse CR Z-score;

## Results

### Identification of a tissue-type independent transcriptional signature of CR in mice

We first analyzed gene expression data from control-fed and CR animals including six published and two unpublished microarray datasets from eight different mouse tissues (S1 Table). We define CR as a regimen of reduced calorie intake in the absence of malnutrition with demonstrated ability to delay aging. Studies not matching this definition, or where actual calorie intake was not documented, were excluded (see Materials and Methods for detailed inclusion criteria). At the level of individual transcripts, we did not identify a single gene that was significantly (*p*<0.05) differentially expressed in all eight tissues. This is not entirely surprising given differences in tissue function and the variation in transcript representation on microarrays over time. Nonetheless, we did identify 272 genes that were significantly (*p*<0.05), and consistently differentially expressed in the majority of tissues (i.e., changes in expression were in the same direction). Among these, there was a clear pattern for increased expression of genes involved in mitochondrial oxidative phosphorylation including *Uqcrh*, *Sdhd*, *Uqcr10*, *Uqcrc2* and *Cox7a2* (S1 Fig.). In addition, there was a trend for decreased expression of pro-inflammatory and immune response genes, including *Ly86*, *Mpeg1*, *C1qb*, *Igh-6*, and *Ccl9*.

**Fig 1:**
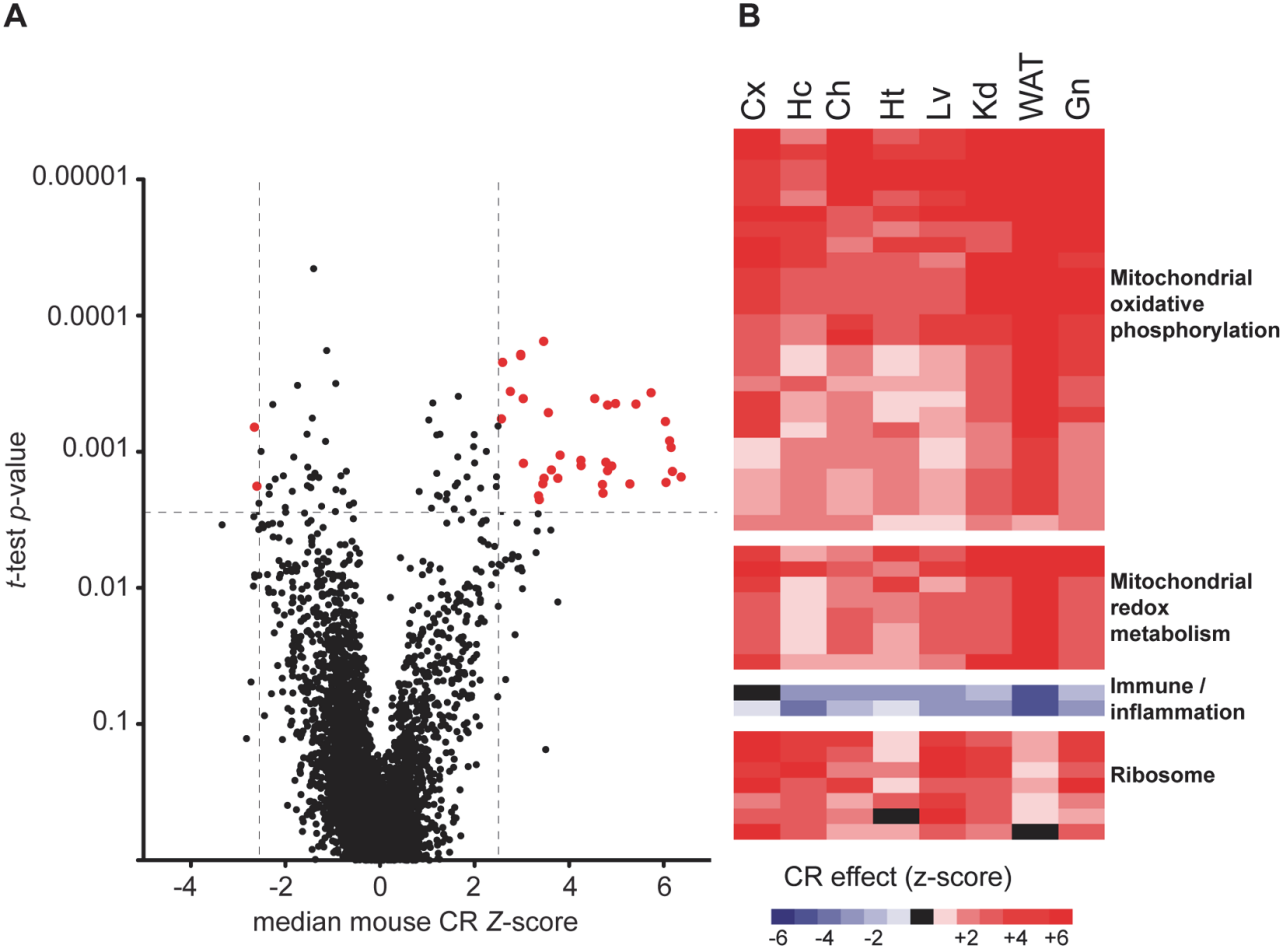
Identification of a multi-tissue transcriptional signature of CR in mice. (*A*) Volcano plot depicting all gene ontology (GO) terms represented across eight mouse tissues. Using the constraints that both FDR adjusted *p*-values were ≤0.00285 and that the median *Z-*score ≥ 2.58 or ≤ -2.58 (up- or downregulated by CR, respectively), we identified 43 GO terms that were significantly modulated by CR across all tissues (red dots). (*B*) Each row indicates one of the 43 GO terms in the CR transcriptional signature, columns indicate the eight mouse tissues studied (Cx = cerebral cortex, Hc = hippocampus, Ch = cochlea, Ht = heart, Lv-liver, Kd = kidney, WAT = epididymal white adipose tissue, Gn = gastrocnemius). The 43 GO terms were clustered into four functional categories including mitochondrial oxidative phosphorylation, mitochondrial redox metabolism, immune response/inflammation and ribosomal structure, and are ordered (top to bottom) as listed in S2 Table. Red or blue fill indicates up- or downregulation by CR, respectively; black fill indicates pathways not changed by treatment (median *Z*-score = 0).

Next we took a broader view, investigating the impact of CR on the activity of gene functional categories. In order to exclude non-specific and ambiguous pathways (e.g., “Metabolism), only those terms from GO Levels 3–5 were considered for analysis; similarly, GO terms annotated with fewer than 10 genes or more than 1,000 genes were excluded from the analysis. A total of 4,949 GO terms were represented in datasets from all eight tissues (Fig. 1*A*). To determine if there were any GO terms significantly modulated across mouse tissues, we applied term-wise one-sample *t*-tests to the PAGE *Z*-scores. We screened for GO terms with false discovery rate (FDR) adjusted *p*-values ≤ 0.00285 (*q*≤0.1) and median *Z*-score ≥ 2.58 or ≤ -2.58; this second constraint assured that at least half of the tissues showed a CR effect on this GO term at *p*<0.001 significance level. Using this approach we identified 43 GO terms that were modulated by CR (Fig. 1*A*, red dots; a detailed list of these pathways is shown in S2 Table). Interestingly, the direction of the effect of CR was consistent across tissues, even though we did not constrain by common direction of change.

Four major themes emerged from this analysis (Fig. 1*B*): the gene sets that were most robustly induced by CR were those involved in mitochondrial structure and oxidative phosphorylation. Reduced expression of nuclear encoded genes involved in mitochondrial oxidative phosphorylation has been reported as a conserved feature of aging in mice and humans [26]. A second category that was upregulated by CR consisted of pathways related to mitochondrial redox metabolism, including carboxylic acid and ketone metabolism. Our data indicate that the mitochondrial response to CR is not limited to the transcripts of the electron transport system but also involves a more broad reprogramming of mitochondrial energy metabolism [3].

The third category identified in the multi-tissue signature of CR included pathways involved in inflammation and the immune response. Consistent with the data for individual genes (S1 Fig.), pathway analysis suggested that CR down-regulates inflammation and immune response pathways relative to control animals. Importantly, CR-repression of these gene sets was detected across tissues and was not limited to tissues associated with protective immune and inflammatory functions. The observation of decreased inflammation and immune response with CR, which protects against multiple age-related diseases and disorders, aligns with the current consensus that elevated inflammatory tone directly contributes to chronic disease [27, 28].

The fourth major category of gene sets induced with CR includes pathways involved in ribosomal structure, suggesting that the ability to synthesize new proteins is preserved in CR tissues. Defects in proteostasis are a common feature of aging and are associated with several neurodegenerative diseases including Alzheimer’s disease and Parkinson’s disease [29] and age-related disorders including sarcopenia [30]. The mechanistic target of rapamycin (mTOR) is a major regulator of ribosomal gene expression. Inhibition of mTOR has been associated with extension of lifespan in mice [31, 32], and reduced signaling through the mTOR pathway has been implicated in the mechanisms of CR in non-mammalian species [33–35]. In these models of extended longevity, inhibition of mTOR signaling is predicted to lower ribosomal gene expression, whereas our data indicate that preservation of at least one aspect of mTOR function (activation of ribosomal gene expression) is associated with delayed aging by CR.

### The multi-tissue response to CR is detected in diverse species and genetic longevity models

**Fig 2:**
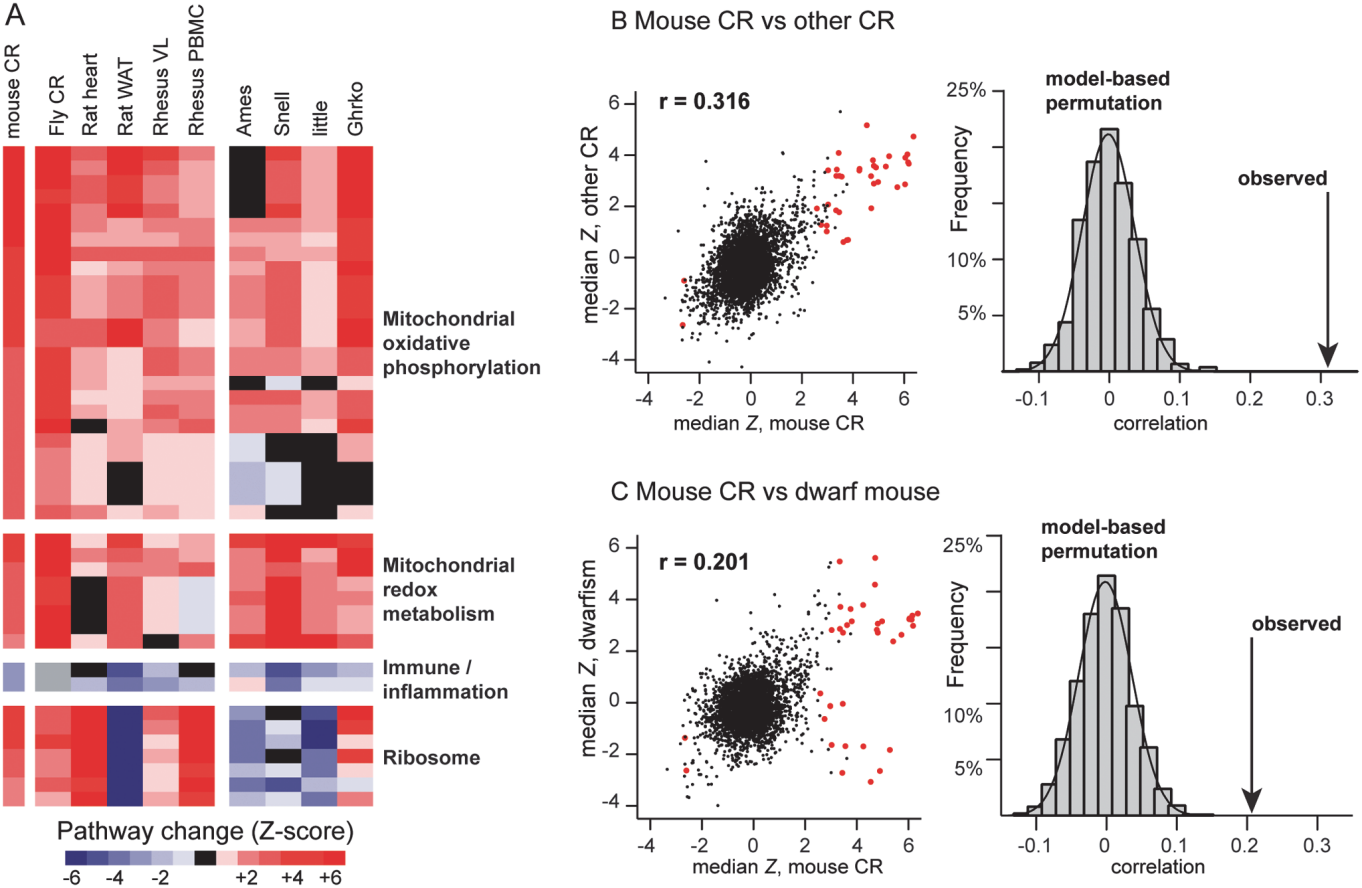
The multi-tissue transcriptional signature of CR in mice is significantly recapitulated in diverse species on CR and in long-lived mouse models. (*A*) The far-left column represents the median Z-score across tissues with GO terms sorted as in Fig. 1 and described in S2 Table. Subsequent columns (left to right) indicate the effect of CR in other species and tissues (*Drosophila*, rat heart, rat white adipose tissue, rhesus monkey *vastus lateralis* muscle and rhesus monkey peripheral blood mononuclear cells), as well as in four long-lived mouse genetic models. Red or blue fill indicates pathways activated or inhibited, respectively, by treatment; black fill indicates pathways not changed by treatment and grey fill indicates pathways not represented in the dataset. (*B*) The observed Spearman correlation between the median *Z*-score for mouse CR and other species subjected to CR (r = 0.316) is greater than would be predicted by model-based (Monte Carlo) permutation (*p* = 0.001), indicating that the overall transcriptional signature of CR in mice is conserved in other species and tissues. (C) The observed Spearman correlation between the median *Z*-score for mouse CR and the median *Z*-score in four mouse models of delayed aging (r = 0.201) is greater than would be predicted by model-based permutation (*p* = 0.001), demonstrating that the overall transcriptional signature of CR in mice is similarly observed in other models of delayed aging. See S1 Table for a detailed description of the individual studies.

We next investigated if the multi-tissue transcriptional signature was present in other species subjected to CR, including *Drosophila*, rat (heart and white adipose tissue), and rhesus monkeys (*vastus lateralis* muscle and peripheral blood mononuclear cells; Fig. 2*A*).

Specifically, we tested the null hypothesis that mouse CR and non-mouse CR tissues would yield independent functional profiles. Using permutation schemes that accounted for GO term overlap (see Materials and Methods), we found that the observed correlation between the mouse CR response (median *Z*-score across eight tissues) and the response to CR in other species was significantly greater than would be predicted by chance (Monte Carlo *p* = 0.001 by permutation test; Spearman coefficient r = 0.316, Fig. 2*B*). These data provide the first quantitative support for the hypothesis that there is an evolutionarily conserved response to CR across *Drosophila*, mice, rats and non-human primates.

To test if the conserved signature of CR was either unique to CR or if elements of the transcriptional program were also evident in other models of extended longevity, we conducted a similar permutation analysis using data from four strains of long-lived dwarf mice with deficiencies in growth hormone/insulin-like growth factor signaling (Ames, Snell, “little”, and GHRKO, ref.[10]). We found that the median *Z*-score across the four dwarf mouse strains was significantly correlated with median mouse CR *Z*-score across tissues (Spearman coefficient r = 0.201, Monte Carlo *p* = 0.001 by permutation test; Fig. 2*C*). While the mitochondrial, metabolic, and inflammatory gene sets were highly correlated between CR and genetically manipulated long-lived animals, there was a lack of agreement for ribosomal gene pathways (Fig. 2A). Nonetheless, the correlations were statistically significant overall, which indicates that there is an evolutionarily conserved response to CR, and that aspects of this program are observed in other models of delayed aging.

### The evolutionarily conserved signature of delayed aging is relevant to disease risk and partially mediated by SIRT3

**Fig 3:**
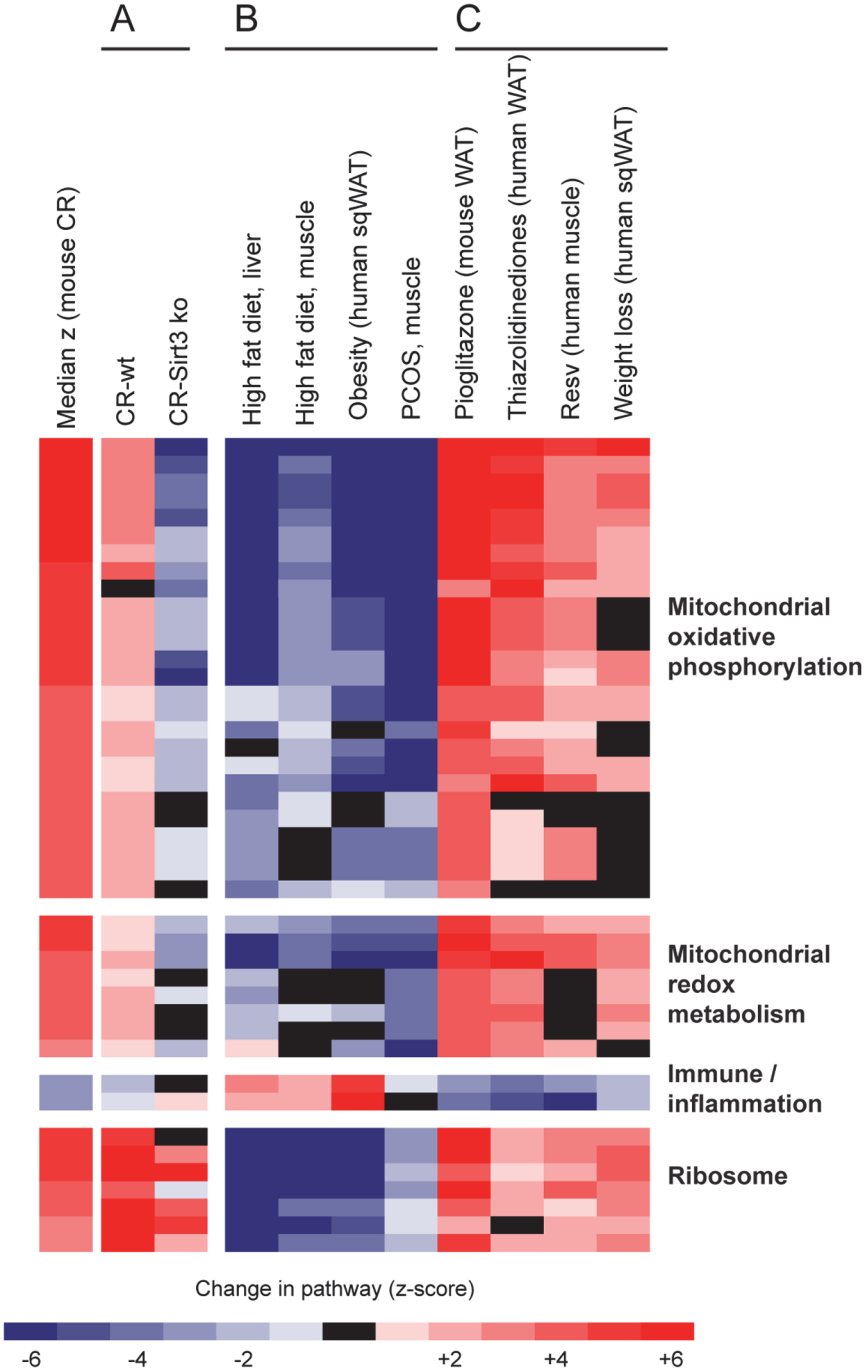
SIRT3 partially mediates the transcriptional signature of delayed aging that is also indicative of risk of disease in mice and humans. Rows indicate GO terms for the conserved signature of delayed aging and are ordered as in Fig. 1 and as described in S2 Table. (*A*) Analysis of gene expression in mouse muscle indicates that the response to CR is seen in wild-type control mice, however absence of the *Sirt3* gene (Sirt3ko) partially blunts the response to CR. (*B*) Consumption of a high fat diet in mice, obesity in humans, and individuals diagnosed with polycystic ovary syndrome (PCOS) display the inverse transcriptional pattern for the longevity signature, whereas (C) interventions that decrease risk of disease (treatment with thiazolidinediones in mice and humans, consumption of resveratrol and weight loss) show an induction of the transcriptional signature of delayed aging. See S1 Table for a detailed description of the individual studies.

We next sought to gain mechanistic insight into what factors may be important in implementing the delayed aging signature. The NAD-dependent deacetylase sirtuin 3 (SIRT3) plays a central role in mitochondrial function [36], and independent studies have implicated SIRT3 in CR [37, 38]. We performed gene expression analysis in gastrocnemius muscle from both age-matched wildtype and SIRT3 knockout mice that have been previously described [38]. In response to CR, the wildtype mice displayed the expected features of the delayed aging signature, however SIRT3 knockout mice fail to induce the mitochondrial and inflammatory components (Fig. 3*A*). The induction of ribosomal pathways was observed in both wildtype and SIRT3 knockout mice, suggesting that the role of SIRT3 may be constrained to the metabolic and anti-inflammatory aspects of CR.

We next asked whether the transcriptional signature of delayed aging was associated with health status outside of the context of aging per se. We searched the NCBI Gene Expression Omnibus repository for datasets reflective of increased disease risk; to maintain consistency with previous analyses, we only selected datasets using the Affymetrix platform. Our analysis of these data revealed that the signature of delayed aging is relevant to metabolic disease profiles (Fig. 3*B*): the inverse of the delayed aging signature is observed in four studies including mice fed a high fat diet [39, 40], obese humans [41] and patients with polycystic ovarian syndrome [15]. Finally, we asked if the signature of delayed aging is induced by interventions that reduce risk of disease, including thiazolidinedione treatment in mice and humans [14], resveratrol consumption in humans [16] and in humans after ~20% weight loss [42]. In all of these studies, our re-analysis indicates that each intervention induced the evolutionarily conserved signature of delayed aging (Fig. 3*C*). These data support the concept that the pathways involved in delayed aging are indicators of disease risk in humans.

## Discussion

We analyzed CR-induced changes in gene expression in eight functionally distinct tissues, each with its own heterogeneous cell population and found a coordinated induction of gene classes involved in mitochondrial energy metabolism. The induction of genes involved in mitochondrial energy metabolism is consistent with the concept of metabolic reprogramming with CR [3, 43]. This component of the delayed aging signature was highly and significantly conserved across species and across models of delayed aging. Importantly, these results provide the first quantitative evidence that altered mitochondrial function is a critical component of slowed aging. The multi-tissue response to CR also included gene classes involved in inflammation that were negatively associated with aging and disease vulnerability. The role of chronic inflammation in disease vulnerability has been long appreciated [27, 44], and decreased expression of inflammatory genes with CR is consistent with the idea that a reduction in inflammatory tone is a key component of aging and associated disease risk [27, 44]. There is a growing appreciation of the interrelationship between immune function and metabolic regulation [27], and further studies will be needed to understand how these processes interact through the aging process.

The relative increase in ribosomal pathway genes with CR touches on a complex issue, the role of TOR in longevity regulation. Our data are not compatible with whole-scale inhibition of TOR function as a means to delay aging. Silencing of the highly repetitive ribosomal locus in yeast was one of the first manipulations shown to extend lifespan [45]. Since then, the TOR signaling pathway has been shown to influence multiple downstream events including lipogenesis, protein turnover, and mitochondrial respiration [46]. The impact of TOR inhibition on metabolism is not static [47] and it is unclear which aspects of TOR signaling are important in delayed mammalian aging. Moreover, while the overall correlation between pathways changed by CR and dwarf mice was statistically significant, the pattern was dissimilar for ribosomal pathways in three of the four models. These data indicate a possible point of departure in mechanisms of lifespan extension in these mouse models from those of CR. One of the key alterations in each of genetic models is a reduction in IGF signaling secondary to their respective primary mutations [48, 49], a physiological change that would be predicted to diminish signaling through mTOR. However, the GHRKO mice are distinct from the other three strains of dwarf mice in that ribosomal gene pathways were induced. The basis for this is not clear, although the fact that CR cannot further enhance longevity in this model, unlike the Ames mouse, suggests a closer alignment of underlying mechanisms for GHRKO and CR [50].

Differences in the selection of datasets may explain why some biological pathways associated with delayed aging were not detected in our analysis. For example, Plank et al. [20] applied a gene-level approach to identify transcripts changed by CR in different tissues and species and found that these candidate genes were associated with lipid metabolism and cholesterol biosynthesis—these pathways were not detected as being modulated by CR in the current study. The authors of the previous analysis report that their analysis was slightly over-represented by datasets from mouse liver, whereas our study included only one dataset for each tissue; this may explain, in part, why lipid and cholesterol metabolism pathways were not identified in our study. Another explanation for the differences between the two studies is that we used a more stringent definition of CR (see Materials and Methods), whereas Plank et al. used a more broad definition of CR and included datasets where the relevance for some the dietary interventions to delayed aging is unclear—for example, studies where food intake was not measured or where the duration of CR was very short (two days). Nonetheless, our initial gene-level analysis is in partial agreement with Plank et al., wherein several lipid metabolism genes tended to be differentially expressed across tissues (including *Hsd17b10*, *Hsd17b11*, *Hsd11b1* and *Fdx1*; S1 Fig.). Plank et al. also report that biological rhythm pathways (“rhythmic process” and “circadian rhythm”) were up-regulated by CR, but our pathway-level analysis did not indicate that these Gene Ontology terms were significantly modulated across tissues in response to CR. However, we did observe that cryptochrome 1 (*Cry1*) was the most over-expressed gene in response to CR in eight mouse tissues (S1 Fig.). *Cry1* plays a key role in regulating circadian rhythmicity and metabolism, such that *Cry*-deficient mice become more obese and develop insulin resistance in response to a high fat diet relative to wild-type controls [51]. Interestingly, *Cry1* and circadian rhythmicity may be related to aging, as increased expression of *Cry1* and more pronounced circadian rhythms were observed in a study of transgenic mice with increased lifespan [52]. While our results do not disagree with the finding of Plank et al., our analysis suggests that transcriptional regulation of the circadian rhythm pathways as a whole may be too variable for detection across tissues. Instead, downstream effects of circadian rhythm activity including mitochondrial activity [53] and inflammation [54] may be more robust indicators of circadian function that relate to delayed aging.

We observed that absence of SIRT3 partially abrogated the ability of CR to activate mitochondrial structure and energy metabolism pathways, consistent with recent advances in the role of sirtuins in metabolic regulation [55]. There is controversy in the field as to whether SIRT3 is exclusively mitochondrial or if specific splice isoforms reside in the nucleus. Our data show that the induction of the transcriptional response to CR is compromised in SIRT3 knockout mice and indicate that, directly or indirectly, SIRT3 regulates the expression of nuclear encoded genes. It is also noteworthy that the anti-inflammatory components of the delayed aging signature are compromised in the SIRT3 null mice. While it is possible that SIRT3 directly regulates the activity of these functional categories, we favor the interpretation that failure to launch the mitochondrial program prohibits effective adaptation of the other components in the CR response. Regardless of the mechanism, there is emerging evidence that SIRT3 plays a key role in regulating aspects of aging and age-related disease: circulating levels of SIRT3 may be a marker of fraility in humans [56], and levels of SIRT3 are decreased in eldery humans in parallel with decreased mitochondrial function [57]. Intriguingly, a recent study suggests that activation of SIRT3 may reverse age-related degeneration of hematopoietic stem cells [58]. Combined with our data showing a blunting of the response to CR in Sirt3 knockout mice, the available data suggest that modulation of SIRT3 is an attractive target for interventions designed to extend healthspan.

Despite the fact that our analysis was conducted on gene expression data generated from different tissues, and from studies employing very different study designs, the potential to translate these insights to human health span is apparent given the reciprocal regulation of the delayed aging signature with obesity and interventions correcting metabolic dysfunction. The evolutionarily conserved signature of delayed aging is relevant to disease risk, as observed by the inverse of the transcriptional response to a high fat diet, obesity and metabolic disease (Fig. 3*B*) as well as the induction of the signature in healthy individuals compared to metabolically compromised individuals (Fig. 3C). All of the latter studies demonstrated a consequent effect at the biochemical and/or physiological level, indicating that the transcriptional response is clinically relevant. Taken together, our data provide quantitative evidence that there is a shared mechanism induced by CR across tissues and species, and this signature is associated with delayed aging and improved health status. We predict that interventions that successfully activate this program hold promise for treatment of age-associated diseases and disorders.

## Supporting Information

S1 Fig Identification of genes differentially expressed in multiple tissues of mice subjected to CR. The transcripts represented on the microarrays differed among experiments such that there were 7,333 genes represented in all eight datasets. Data shown represent the top 10% of up regulated and down regulated genes out of the 272 genes that were differentially expressed in 5/8 mouse tissues (p<0.05). Expression level in control-fed mice can be considered to be “1”; the fold change value indicated in the figure represents the change in expression of that gene in response to CR. Data were ranked by cumulative fold change across all tissues. Left to right: cerebral cortex, hippocampus, cochlea, heart, liver, kidney, white adipose tissue and gastrocnemius (n = 5 mice per group/tissue except for cochlea where n = 3 mice per group). Fold change in expression is indicated by yellow (up) or cyan (down).
(TIF)

S1 Table Detailed description of the microarray datasets analyzed in the present study. This includes the accession number for retrieving the raw data ("Data source") as well as the PubMed identifier (PMID) for the first published study describing the microarray data.
(XLSX)

S2 Table Gene Ontology pathways representing the evolutionarily conserved transcriptional signature of delayed aging. CR Z-score represents the median Z-score for each pathway across all eight tissues from CR mice. An FDR-adjusted p-value <0.00285 assures that at least half of the tissues have a CR effect <0.001. The order (top to bottom) in which these pathways appear in this table reflects their order in Figs. 1–3 of the main text.
(XLSX)

S3 Table ARRIVE checklist. The ARRIVE Guidelines Checklist.
(PDF)

## References

[1] Hayflick L (2007) Biological aging is no longer an unsolved problem. Annals of the New York Academy of Sciences 1100:1–13. 17460161 10.1196/annals.1395.001

[2] Lopez-Otin C, Blasco MA, Partridge L, Serrano M, Kroemer G (2013) The hallmarks of aging. Cell 153(6):1194–1217. 10.1016/j.cell.2013.05.039 23746838 PMC3836174

[3] Anderson RM, Weindruch R (2010) Metabolic reprogramming, caloric restriction and aging. Trends Endocrinol Metab 21(3):134–141. 10.1016/j.tem.2009.11.005 20004110 PMC2831168

[4] Colman RJ, Anderson RM, Johnson SC, Kastman EK, Kosmatka KJ, Beasley TM et al (2009) Caloric restriction delays disease onset and mortality in rhesus monkeys. Science 325(5937):201–204. 10.1126/science.1173635 19590001 PMC2812811

[5] Cao SX, Dhahbi JM, Mote PL, Spindler SR (2001) Genomic profiling of short- and long-term caloric restriction effects in the liver of aging mice. Proc Natl Acad Sci U S A 98(19):10630–10635. 11535822 10.1073/pnas.191313598 PMC58517

[6] Edwards MG, Anderson RM, Yuan M, Kendziorski CM, Weindruch R, Prolla TA (2007) Gene expression profiling of aging reveals activation of a p53-mediated transcriptional program. BMC Genomics 8:80 17381838 10.1186/1471-2164-8-80 PMC1847444

[7] Lee CK, Allison DB, Brand J, Weindruch R, Prolla TA (2002) Transcriptional profiles associated with aging and middle age-onset caloric restriction in mouse hearts. Proc Natl Acad Sci U S A 99(23):14988–14993. 12419851 10.1073/pnas.232308999 PMC137532

[8] Lee CK, Weindruch R, Prolla TA (2000) Gene-expression profile of the ageing brain in mice. Nat Genet 25(3):294–297. 10888876 10.1038/77046

[9] Anderson RM, Weindruch R (2012) The caloric restriction paradigm: implications for healthy human aging. Am J Hum Biol 24(2):101–106. 10.1002/ajhb.22243 22290875 PMC3705772

[10] Barger JL, Walford RL, Weindruch R (2003) The retardation of aging by caloric restriction: its significance in the transgenic era. Exp Gerontol 38(11–12):1343–1351. 14698815 10.1016/j.exger.2003.10.017

[11] Brown-Borg HM, Borg KE, Meliska CJ, Bartke A (1996) Dwarf mice and the ageing process. Nature 384(6604):33 8900272 10.1038/384033a0

[12] Flurkey K, Papaconstantinou J, Miller RA, Harrison DE (2001) Lifespan extension and delayed immune and collagen aging in mutant mice with defects in growth hormone production. Proc Natl Acad Sci U S A 98(12):6736–6741. 11371619 10.1073/pnas.111158898 PMC34422

[13] Coschigano KT, Clemmons D, Bellush LL, Kopchick JJ (2000) Assessment of growth parameters and life span of GHR/BP gene-disrupted mice. Endocrinology 141(7):2608–2613. 10875265 10.1210/endo.141.7.7586

[14] Sears DD, Hsiao G, Hsiao A, Yu JG, Courtney CH, Ofrecio CH et al. (2009) Mechanisms of human insulin resistance and thiazolidinedione-mediated insulin sensitization. Proc Natl Acad Sci U S A 106(44):18745–18750. 10.1073/pnas.0903032106 19841271 PMC2763882

[15] Skov V, Glintborg D, Knudsen S, Tan Q, Jensen T, Kruse TA et al (2008) Pioglitazone enhances mitochondrial biogenesis and ribosomal protein biosynthesis in skeletal muscle in polycystic ovary syndrome. PLoS One 3(6):e2466 10.1371/journal.pone.0002466 18560589 PMC2413008

[16] Timmers S, Konings E, Bilet L, Houtkooper RH, van de Weijer T, Goossense GH et al (2011) Calorie restriction-like effects of 30 days of resveratrol supplementation on energy metabolism and metabolic profile in obese humans. Cell Metab 14(5):612–622. 10.1016/j.cmet.2011.10.002 22055504 PMC3880862

[17] Tome-Carneiro J, Gonzalvez M, Larrosa M, Yanez-Gascon MJ, Garcia-Almagro FJ, Ruiz-Ros JA et al (2013) Grape resveratrol increases serum adiponectin and downregulates inflammatory genes in peripheral blood mononuclear cells: a triple-blind, placebo-controlled, one-year clinical trial in patients with stable coronary artery disease. Cardiovascular drugs and therapy / sponsored by the International Society of Cardiovascular Pharmacotherapy 27(1):37–48. 10.1007/s10557-012-6427-8 23224687 PMC3555235

[18] Swindell WR (2009) Genes and gene expression modules associated with caloric restriction and aging in the laboratory mouse. BMC Genomics 10:585 10.1186/1471-2164-10-585 19968875 PMC2795771

[19] Swindell WR (2008) Comparative analysis of microarray data identifies common responses to caloric restriction among mouse tissues. Mechanisms of ageing and development 129(3):138–153. 18155270 10.1016/j.mad.2007.11.003 PMC2702675

[20] Plank M, Wuttke D, van Dam S, Clarke SA, de Magalhaes JP (2012) A meta-analysis of caloric restriction gene expression profiles to infer common signatures and regulatory mechanisms. Molecular bioSystems 8(4):1339–1349. 10.1039/c2mb05255e 22327899

[21] Gould GW, Holman GD (1993) The glucose transporter family: structure, function and tissue-specific expression. The Biochemical journal 295 (Pt 2):329–341. 8240230 10.1042/bj2950329 PMC1134886

[22] Pearson KJ, Baur JA, Lewis KN, Peshkin L, Price NL, Labinskyy N et al (2008) Resveratrol delays age-related deterioration and mimics transcriptional aspects of dietary restriction without extending life span. Cell Metab 8(2):157–168. 10.1016/j.cmet.2008.06.011 18599363 PMC2538685

[23] Pugh TD, Klopp RG, Weindruch R (1999) Controlling caloric consumption: protocols for rodents and rhesus monkeys. Neurobiol Aging 20(2):157–165. 10537025 10.1016/s0197-4580(99)00043-3

[24] Kim SY, Volsky DJ (2005) PAGE: parametric analysis of gene set enrichment. BMC Bioinformatics 6:144 15941488 10.1186/1471-2105-6-144 PMC1183189

[25] Newton MA, Quintana FA, den Boon JA, Senguta S, Ahlquist P (2007) Random-set methods identify distinct aspects of the enrichment signal in gene-set analysis. Annals of Applied Statistics 1(1):85–106.

[26] Zahn JM, Sonu R, Vogel H, Crane E, Mazan-Mamczarz K, Rabkin R et al (2006) Transcriptional profiling of aging in human muscle reveals a common aging signature. PLoS Genet 2(7):e115 16789832 10.1371/journal.pgen.0020115 PMC1513263

[27] Hotamisligil GS (2006) Inflammation and metabolic disorders. Nature 444(7121):860–867. 17167474 10.1038/nature05485

[28] Osborn O, Olefsky JM (2012) The cellular and signaling networks linking the immune system and metabolism in disease. Nature medicine 18(3):363–374. 10.1038/nm.2627 22395709

[29] Powers ET, Morimoto RI, Dillin A, Kelly JW, Balch WE (2009) Biological and chemical approaches to diseases of proteostasis deficiency. Annual review of biochemistry 78:959–991. 10.1146/annurev.biochem.052308.114844 19298183

[30] Saini A, Faulkner S, Al-Shanti N, Stewart C (2009) Powerful signals for weak muscles. Ageing research reviews 8(4):251–267. 10.1016/j.arr.2009.02.001 19716529

[31] Harrison DE, Strong R, Sharp ZD, Nelson JF, Astle CM, Flurkey K et al (2009) Rapamycin fed late in life extends lifespan in genetically heterogeneous mice. Nature 460(7253):392–395. 10.1038/nature08221 19587680 PMC2786175

[32] Selman C, Tullet JM, Wieser D, Irvine E, Lingard SJ, Choudhury AI et al (2009) Ribosomal protein S6 kinase 1 signaling regulates mammalian life span. Science 326(5949):140–144. 10.1126/science.1177221 19797661 PMC4954603

[33] Bonawitz ND, Chatenay-Lapointe M, Pan Y, Shadel GS (2007) Reduced TOR signaling extends chronological life span via increased respiration and upregulation of mitochondrial gene expression. Cell Metab 5(4):265–277. 17403371 10.1016/j.cmet.2007.02.009 PMC3460550

[34] Kaeberlein M, Powers RW, Steffen KK, Westman EA, Hu D, Dang N et al (2005) Regulation of yeast replicative life span by TOR and Sch9 in response to nutrients. Science 310(5751):1193–1196. 16293764 10.1126/science.1115535

[35] Zid BM, Rogers AN, Katewa SD, Vargas MA, Kolipinski MC, Lu TA et al (2009) 4E-BP extends lifespan upon dietary restriction by enhancing mitochondrial activity in Drosophila. Cell 139(1):149–160. 10.1016/j.cell.2009.07.034 19804760 PMC2759400

[36] Huang JY, Hirschey MD, Shimazu T, Ho L, Verdin E (2010) Mitochondrial sirtuins. Biochimica et biophysica acta 1804(8):1645–1651. 10.1016/j.bbapap.2009.12.021 20060508

[37] Qiu X, Brown K, Hirschey MD, Verdin E, Chen D (2010) Calorie restriction reduces oxidative stress by SIRT3-mediated SOD2 activation. Cell Metab 12(6):662–667. 10.1016/j.cmet.2010.11.015 21109198

[38] Someya S, Yu W, Hallows WC, Xu J, Vann JM, Leeuwenburgh C et al (2010) Sirt3 mediates reduction of oxidative damage and prevention of age-related hearing loss under caloric restriction. Cell 143(5):802–812. 10.1016/j.cell.2010.10.002 21094524 PMC3018849

[39] de Wilde J, Smit E, Mohren R, Boekschoten MV, de Groot P, van den Berg SA et al (2009) An 8-week high-fat diet induces obesity and insulin resistance with small changes in the muscle transcriptome of C57BL/6J mice. Journal of nutrigenetics and nutrigenomics 2(6):280–291. 10.1159/000308466 20588053

[40] Hirschey MD, Shimazu T, Jing E, Grueter CA, Collins AM, Aouizerat B et al (2011) SIRT3 deficiency and mitochondrial protein hyperacetylation accelerate the development of the metabolic syndrome. Molecular cell 44(2):177–190. 10.1016/j.molcel.2011.07.019 21856199 PMC3563434

[41] Arner E, Mejhert N, Kulyte A, Balwierz PJ, Pachkov M, Cormont M, et al (2012) Adipose tissue microRNAs as regulators of CCL2 production in human obesity. Diabetes 61(8):1986–1993. 10.2337/db11-1508 22688341 PMC3402332

[42] Johansson LE, Danielsson AP, Parikh H, Klintenberg M, Norstrom F, Groop L et al (2012) Differential gene expression in adipose tissue from obese human subjects during weight loss and weight maintenance. The American journal of clinical nutrition 96(1):196–207. 10.3945/ajcn.111.020578 22648723

[43] Guarente L (2008) Mitochondria—a nexus for aging, calorie restriction, and sirtuins? Cell 132(2):171–176. 10.1016/j.cell.2008.01.007 18243090 PMC2680180

[44] Shoelson SE, Lee J, Goldfine AB (2006) Inflammation and insulin resistance. J Clin Invest 116(7):1793–1801. 16823477 10.1172/JCI29069 PMC1483173

[45] Kaeberlein M, McVey M, Guarente L (1999) The SIR2/3/4 complex and SIR2 alone promote longevity in Saccharomyces cerevisiae by two different mechanisms. Genes Dev 13(19):2570–2580. 10521401 10.1101/gad.13.19.2570 PMC317077

[46] Laplante M, Sabatini DM (2012) mTOR signaling in growth control and disease. Cell 149(2):274–293. 10.1016/j.cell.2012.03.017 22500797 PMC3331679

[47] Fang Y, Westbrook R, Hill C, Boparai RK, Arum O, Spong A et al (2013) Duration of rapamycin treatment has differential effects on metabolism in mice. Cell Metab 17(3):456–462. 10.1016/j.cmet.2013.02.008 23473038 PMC3658445

[48] Bartke A, Sun LY, Longo V (2013) Somatotropic signaling: trade-offs between growth, reproductive development, and longevity. Physiological reviews 93(2):571–598. 10.1152/physrev.00006.2012 23589828 PMC3768106

[49] Berryman DE, Christiansen JS, Johannsson G, Thorner MO, Kopchick JJ (2008) Role of the GH/IGF-1 axis in lifespan and healthspan: lessons from animal models. Growth hormone & IGF research: official journal of the Growth Hormone Research Society and the International IGF Research Society 18(6):455–471. 10.1016/j.ghir.2008.05.005 PMC2631405 18710818

[50] Bartke A, Westbrook R (2012) Metabolic characteristics of long-lived mice. Frontiers in genetics 3:288 10.3389/fgene.2012.00288 23248643 PMC3521393

[51] Barclay JL, Shostak A, Leliavski A, Tsang AH, Johren O, Muller-Fielitz H et al (2013) High-fat diet-induced hyperinsulinemia and tissue-specific insulin resistance in Cry-deficient mice. American journal of physiology. Endocrinology and metabolism 304(10):E1053–1063. 10.1152/ajpendo.00512.2012 23531614

[52] Froy O, Chapnik N, Miskin R (2006) Long-lived alphaMUPA transgenic mice exhibit pronounced circadian rhythms. American journal of physiology. Endocrinology and metabolism 291(5):E1017–1024. 16787960 10.1152/ajpendo.00140.2006

[53] Peek CB, Affinati AH, Ramsey KM, Kuo HY, Yu W, Sena LA et al (2013) Circadian clock NAD+ cycle drives mitochondrial oxidative metabolism in mice. Science 342(6158):1243417 10.1126/science.1243417 24051248 PMC3963134

[54] Oliva-Ramirez J, Moreno-Altamirano MM, Pineda-Olvera B, Cauich-Sanchez P, Sanchez-Garcia FJ (2014) Crosstalk between circadian rhythmicity, mitochondrial dynamics and macrophage bactericidal activity. Immunology 143(3):490–497. 10.1111/imm.12329 24903615 PMC4212961

[55] Nogueiras R, Habegger KM, Chaudhary N, Finan B, Banks AS, Dietrich MO et al. (2012) Sirtuin 1 and sirtuin 3: physiological modulators of metabolism. Physiological reviews 92(3):1479–1514. 10.1152/physrev.00022.2011 22811431 PMC3746174

[56] Kumar R, Mohan N, Upadhyay AD, Singh AP, Sahu V, Dwivedi S et al. (2014) Identification of serum sirtuins as novel noninvasive protein markers for frailty. Aging cell 13(6):975–980. 10.1111/acel.12260 25100619 PMC4326933

[57] Joseph AM, Adhihetty PJ, Buford TW, Wohlgemuth SE, Lees HA, Nguyen LM et al. (2012) The impact of aging on mitochondrial function and biogenesis pathways in skeletal muscle of sedentary high- and low-functioning elderly individuals. Aging cell 11(5):801–809. 10.1111/j.1474-9726.2012.00844.x 22681576 PMC3444680

[58] Brown K, Xie S, Qiu X, Mohrin M, Shin J, Liu Y et al. (2013) SIRT3 reverses aging-associated degeneration. Cell reports 3(2):319–327. 10.1016/j.celrep.2013.01.005 23375372 PMC3582834

