# Supplementary figures and images for: A Conserved Transcriptional Signature of Delayed Aging and Reduced Disease Vulnerability Is Partially Mediated by SIRT3
Source: PLoS One. 2015 Apr 1;10(4):e0120738. doi: 10.1371/journal.pone.0120738 (PMC4382298; doi:10.1371/journal.pone.0120738)

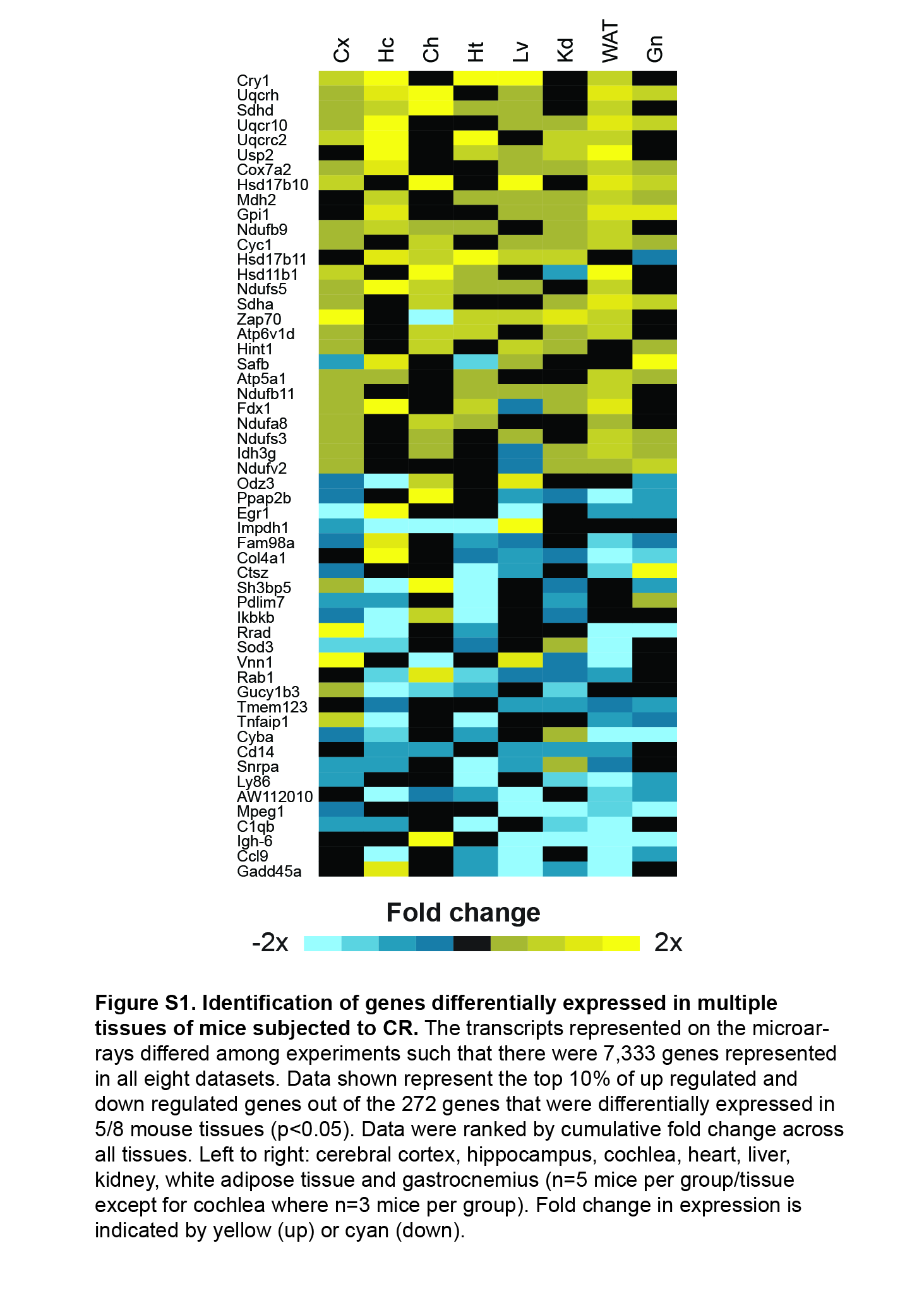

Supplement: S1 Fig — The transcripts represented on the microarrays differed among experiments such that there were 7,333 genes represented in all eight datasets. Data shown represent the top 10% of up regulated and down regulated genes out of the 272 genes that were differentially expressed in 5/8 mouse tissues (p<0.05). Expression level in control-fed mice can be considered to be “1”; the fold change value indicated in the figure represents the change in expression of that gene in response to CR. Data were ranked by cumulative fold change across all tissues. Left to right: cerebral cortex, hippocampus, cochlea, heart, liver, kidney, white adipose tissue and gastrocnemius (n = 5 mice per group/tissue except for cochlea where n = 3 mice per group). Fold change in expression is indicated by yellow (up) or cyan (down). (TIF) [file pone.0120738.s001.tif]
